# Rare Case of Duodenal Metastasis From Pulmonary Squamous Cell Carcinoma

**DOI:** 10.1177/2324709617737567

**Published:** 2017-10-25

**Authors:** Zain Memon, Samson Ferm, Constantine Fisher, Akil Hassam, Jean Luo, Sang Hoon Kim

**Affiliations:** 1 Northwell Health Lenox Hill Hospital, New York, NY, USA; 2 New York Presbyterian Queens, Queens, NY, USA

**Keywords:** colon, small bowel, malignancy, squamous cell carcinoma, colonoscopy, endoscopy

## Abstract

Pulmonary squamous cell carcinoma is the second most common non–small cell malignancy of the lung. It commonly metastasizes to the adrenal glands, bone, liver, brain, and kidneys. Most occurrences of metastatic squamous cell carcinoma involving the gastrointestinal tract originate from primary lung tumors. Metastasis to the duodenum, however, is exceedingly rare, with very few cases of stomach or duodenal involvement described in the literature. We report the case of a patient with stage IV pulmonary squamous cell carcinoma metastasizing to the duodenum with an uncommon presentation to add to the paucity of literature available regarding this rare finding.

## Case Report

**Figure 1.**
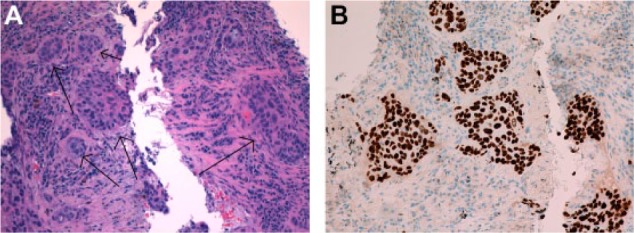
(A) Lung tissue procured from CT-guided biopsy showing poorly differentiated and malignant appearing solid cell nests (arrows). (B) Staining of the lung tissue demonstrated diffuse positivity for p63, consistent with squamous cell carcinoma.

An 81-year-old Caucasian male was referred to the emergency department by his oncologist for symptomatic anemia. The patient had a past medical history of hypertension, diabetes mellitus type 2, and a 20 pack-year history of tobacco use. The patient was in a normal state of health until 1 month prior to presentation, when he began experiencing pleuritic chest pain, shortness of breath, cough, and generalized weakness. Due to an extensive tobacco use history, a screening low-resolution computed tomography (CT) scan was ordered. This revealed a right upper lung mass, measuring 5.5 cm, extending into the mediastinum and precarinal region with extensive lymphadenopathy. Hypodense hepatic lesions were also seen in the right and left hepatic lobes, with the largest lesion measuring 4 cm. This was suspicious for metastasis. Patient underwent CT-guided core biopsy (Figure 1). Immunohistochemistry demonstrated diffuse positivity for p63 and was negative for thyroid transcription factor 1 (TTF-1). The patient was diagnosed with reported squamous cell carcinoma (SCC) of the lung and was scheduled to begin palliative chemotherapy as an outpatient.

On the day of presentation, the patient complained of 1 week of melena, shortness of breath, and generalized weakness. He denied abdominal pain, nausea, hematemesis, and hematochezia. In the emergency department, he was tachycardic and hypotensive. Physical exam was remarkable for conjunctival and gingival pallor, poor capillary refill, and orthostasis. Rectal exam showed grossly brown stool which tested hemoccult positive. Laboratory tests revealed a hemoglobin of 5.8 g/dL, a hematocrit of 19.1 g/dL, a mean corpuscular volume of 85.3 fL, a platelet count of 125 000/µL, a blood urea nitrogen of 26 mg/dL, and a creatinine of 0.9 mg/dL. Coagulation panel showed partial thromboplastin time of 14.4, international normalized ratio of 1.28, and an activated partial thromboplastin time of 23.4.

**Figure 2.**
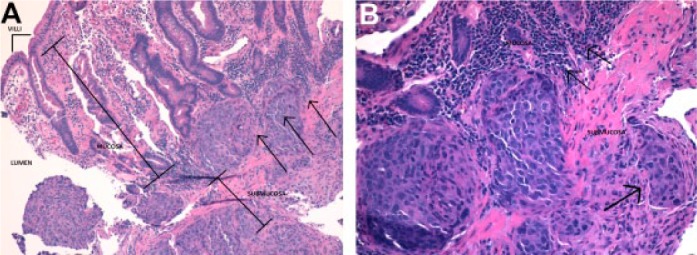
(A) Dysplastic duodenal muscosa with squamous cell carcinoma invading from the submucosa (arrows). (B) Tumor cells invading the muscularis mucosa and small vascular invasion (arrows).

The patient was transfused 1 unit of packed red blood cells. He was started on intravenous proton pump inhibitor infusion and was scheduled for an esophagogastroduodenoscopy. The esophagogastroduodenoscopy detected mild bulbar erythema with erosions but no active bleeding. The patient underwent CT angiography, which identified a bleeding source in the distal duodenum; therefore, the decision was made to forego colonoscopy and proceed to capsule endoscopy (CE). On CE, bright red blood was noted in the distal duodenum. The patient then underwent double balloon enteroscopy. An actively bleeding, infiltrating, fungating mass was visualized in the fourth part of the duodenum. Biopsy of the mass with hematoxylin and eosin stained sections showed mitosis, nuclear polymorphism, poorly differentiated cells in nests and cords, and vascular invasion. Immunohistochemical analysis was consistent with SCC (Figure 2). Given this finding and the result of the low-resolution CT scan, the duodenal lesion was deemed a metastasis of SCC from a primary pulmonary malignancy. The patient’s hemoglobin remained stable, and palliative radiation and chemotherapy was initiated.

## Discussion

Non–small cell lung cancers typically spread to the bone, brain, adrenal glands, and/or kidneys.^1^ Gastrointestinal metastasis is exceedingly rare. In a review of literature from 1961 to 2003, Hillenbrand et al found 58 documented cases.^2^ The most common histological subtypes were squamous cell, followed by large cell and adenocarcinoma.^2^ Multiple case reports describe the spread of adenocarcinoma to the duodenum.^1,3,4^ To our knowledge, only a few cases of pulmonary squamous cell carcinoma (PSCC) metastasis to the small bowel have been described with spread to the jejunum reported 50.9%, ileum 33.3%, and duodenum 15.8%.^4-9^

The most common presenting symptoms as per previous case reports are abdominal pain, cramping, nausea, vomiting, weight loss, and constipation. This case is notable for the absence of these symptoms, with the main presenting feature being symptomatic anemia and melena.^10-13^

Due to the low incidence of malignancies, both primary and secondary, within the small bowel, diagnosis can be challenging. A high level of suspicion is required in a patient with known malignancy who presents with anemia, upper gastrointestinal bleeding, or an acute abdomen with pneumoperitoneum found on radiography. CT scanning and small bowel follow through were found to have low sensitivity in identifying metastatic melanoma.^14^

Capsule endoscopy has been shown to detect more small bowel irregularities than push enteroscopy and has become first line in the diagnosis of obscure gastrointestinal bleeding. However, in one study, out of 150 CEs performed, 3 tumors were undetected. The authors concluded that double-balloon enteroscopy should be used in patients with suspected bleeding in the small bowel.^15^ In another report, technetium-labelled red blood cells were used successfully to identify the bleeding site due to a small bowel metastasis from lung primary.^13^

Once tissue is obtained, immunohistochemical markers are widely used to distinguish between small cell lung carcinoma and poorly differentiated PSCC. In a review of literature, 13 poorly differentiated PSCCs expressed p63 and did not express TTF-1.^16^ Immunoreactivity for p63 was also noted in bronchial reserve cells and metaplastic squamous cells. TTF-1 is commonly used to identify adenocarcinoma, whereas p63, CK5/6, and 34βE12 are often expressed in SCC.^16^ Likewise, immunohistochemical staining demonstrated diffuse positivity for p63 and was negative for TTF-1, consistent with SCC.

Identification of metastatic or primary SCC in the duodenum is an important consideration to make when addressing prognosis and treatment options. It may be difficult to establish a clear diagnosis between primary SCC and metastatic SCC based on vague symptoms and imaging findings; however, clinical diagnosis can be achieved through tissue biopsy and immunohistochemical staining of the tumor in relation to the primary tumor. The pathogenesis of SCC in the gastrointestinal tract has not been well-elucidated, with several theorizing direct invasion, intraperitoneal dissemination, and/or lymphatic or hematogenous cancer spread.^5-7^ Several theories regarding the origin of SCC in the duodenum have been proposed, including nests of ectopic squamous cells, the proliferation of uncommitted mucosal basal cells into squamous cells, squamous metaplasia secondary to chronic mucosal damage, squamous differentiation in a preexisting adenocarcinoma, and multipotent stem cells in the gastrointestinal mucosa.^5-7^

In our presentation, the patient was found to have several more common hepatic lesions on CT that might extend directly to the adjacent duodenum; however, it would be premature to dismiss the idea of a truly bona fide duodenal metastasis from a primary pulmonary malignancy based on tissue biopsies and immunohistochemical staining of the mass.

Prognosis is poor in patients with metastatic disease of the small bowel. This is likely attributable to lack of symptoms until later stages of disease. With the increasing use of wireless CE and double balloon enteroscopy, diagnosis and management of metastatic lesions of the small bowel can improve. In a patient who presents with abdominal symptoms in the setting of lung cancer, metastasis to the gastrointestinal tract may be considered.

The main treatment goal is to control bleeding based on the site and size of duodenal involvement. If the mass is found to be <1 cm in size, endoscopic resection can serve as a viable option. However, with increasing size, surgical options like duodenectomy and pancreatoduodenectomy might relieve symptoms without altering prognosis.^7^ Morris et al^17^ reported a mean survival time of 2 months after detection of gastrointestinal tract metastasis in 8 patients, and Wiedemer et al^18^ recorded a mean survival time of 4 months. In previous case studies, only 1 patient, described in Hinoshita et al, survived for 1 year postoperatively. Early resection may prevent further complications such as gastrointestinal perforation, obstruction, and hemorrhage. Treatment protocol includes induction chemotherapy, followed by surgical resection, and then adjunctive chemotherapy.^5-8^ Irradiation of gut can lead to sequelea of other complications. Some reports have indicated increased risk of perforation with chemotherapy and radiotherapy treatments.^19,20^ It is important to understand that with metastases to multiple organs and other serious complications, treatment is only palliative.

The prevalence of PSCC metastasizing to the gastrointestinal tract is exceedingly rare. Although treatment options are available, mostly palliative, the complications remain. Early identification of metastatic spread will make it easier to cater treatment options specific to patient clinical symptoms, immunohistochemical analysis, and lastly, prognosis.
